# Quercetin Protects against Cadmium-Induced Renal Uric Acid Transport System Alteration and Lipid Metabolism Disorder in Rats

**DOI:** 10.1155/2012/548430

**Published:** 2012-05-29

**Authors:** Ju Wang, Ying Pan, Ye Hong, Qing-Yu Zhang, Xiao-Ning Wang, Ling-Dong Kong

**Affiliations:** Key Laboratory of Pharmaceutical Biotechnology, School of Life Sciences, Nanjing University, Nanjing 210093, China

## Abstract

Hyperuricemia and dyslipidemia are involved in Cd nephrotoxicity. The aim of this study was to determine the effect of quercetin, a dietary flavonoid with anti-hyperuricemic and anti-dyslipidemic properties, on the alteration of renal UA transport system and disorder of renal lipid accumulation in 3 and 6 mg/kg Cd-exposed rats for 4 weeks. Cd exposure induced hyperuricemia with renal XOR hyperactivity and UA excretion dysfunction in rats. Simultaneously, abnormal expression levels of renal UA transport-related proteins including RST, OAT1, MRP4 and ABCG2 were observed in Cd-exposed rats with inhibitory activity of renal Na^+^-K^+^-ATPase. Furthermore, Cd exposure disturbed lipid metabolism with down-regulation of AMPK and its downstream targets PPAR**α**, OCTN2 and CPT1 expressions, and up-regulation of PGC-1**β** and SREBP-1 expressions in renal cortex of rats. We had proved that Cd-induced disorder of renal UA transport and production system might have cross-talking with renal AMPK-PPAR**α**/PGC-1**β** signal pathway impairment, contributing to Cd nephrotoxicity of rats. Quercetin was found to be effective against Cd-induced dysexpression of RST and OAT1 with XOR hyperactivity and impairment of AMPK-PPAR**α**/PGC-1**β** signal pathway, resulting in renal lipid accumulation reduction of rats.

## 1. Introduction

Cadmium (Cd) is considered to be toxic, heavy metal that causes nephrotoxicity in humans [1–3]. More evidence demonstrates the role of high-serum uric acid (UA) levels in Cd-induced overproduction of endogenous reactive oxygen species (ROS), which subsequently leads to renal injury [4, 5] and lipid metabolism disorder [6]. Xanthine oxidoreductase (XOR), including its initial form xanthine dehydrogenase (XDH, EC1.1.1.204) and xanthine oxidase (XO, EC1.2.3.2), is the key enzyme to catalyze UA production. Cd exposure induces the conversion of XDH into XO [7] and causes XO activation [8]. Renal organic ion transporters of solute carrier (*SLC*) 22 family are increasingly recognized as important determinants of urate transport. Urate transporter 1 (URAT1, *SLC22A12*) is the major absorptive urate transport protein in the kidney being responsible for regulation of blood urate homeostasis [9]. In addition to URAT1, OAT1 (*SLC22A6*) is a basolateral urate transporter [9]. The efflux transporters of the ATP binding cassette (*ABC*) family such as the multidrug resistance protein 4 (MRP4, *ABCC4*) [10] and breast cancer-resistance protein (BCRP, *ABCG2*) [11] seem to be major candidates for urate secretory transport. Therefore, abnormality of these renal organic ion transporters may contribute to the impaired UA excretion and hyperuricemia [9–12].

As important cross-regulators, UA and XOR are directly or indirectly related to lipid metabolism [13]. Dyslipidemia is suggested to be responsible for the progression of chronic kidney disease [14]. Cd exposure can alter serum lipid level and liver lipid metabolism in male Wistar rats [15] and induce lipid accumulation in the tubular lumen of male cat [16]. Therefore, animal studies evaluating Cd exposure-induced dysfunction of renal UA transport and production system are needed to verify its role in lipid metabolism disorder in Cd nephrotoxicity.

A dietary flavonoid quercetin from herbal foods has a variety of biological activities [17, 18]. Our previous studies have demonstrated that quercetin regulated renal UA transport-related proteins in fructose-induced hyperuricemic rats [19] and reduced hepatic XOR hyperactivity in potassium oxonate-induced hyperuricemic mice [20], being an effective antihyperuricemic agent. Moreover, quercetin enhances lipid metabolism in triton-fed rats [21] and inhibits proinflammatory factors against Cd-induced nephrotoxicity [22]. However, the efficacy of quercetin for hyperuricemia and lipid accumulation involved in Cd nephrotoxicity has not been investigated so far.

Therefore, the present study aimed to explain the effects of Cd exposure on renal UA transport-related proteins including renal-specific transporter (RST, a homolog of hURAT1, identified in rats), OAT1, MRP4, and ABCG2 as well as XOR activity in rats. We also investigated its effects on the expression levels of lipid metabolism-related genes including renal AMP-activated protein kinase (AMPK), its downstream targets peroxisome proliferator-activated receptor *α* (PPAR*α*), organic cation transporter 2 (OCTN2), carnitine palmityl transferase 1 (CPT1), PPAR*γ* coactivators 1*β* (PGC-1*β*), and sterol regulatory element-binding protein 1 (SREBP-1) in rats, demonstrating renal lipid metabolism disorder involved in renal UA transport system dysregulation and XOR hyperactivity in Cd nephrotoxicity of rats. Furthermore, we evaluated the efficacy of quercetin treatment in ameliorating hyperuricemia and lipid accumulation in Cd-exposed rats and explored its mechanisms.

## 2. Materials and Methods

### 2.1. Materials

Cadmium chloride (CdCl_2_, AR) and quercetin were obtained from Sigma-Aldrich (St. Louis, MO, USA). Diagnostic kits for the activity or level of n-acetyl-*β*-glucosaminidase (NAG), Na^+^-K^+^-ATPase, protein, albumin (ALB), creatinine (Cr), and triglyceride (TG) were obtained from Jiancheng Biotech Institution (Nanjing, China). The enzyme-linked immunosorbent assay (ELISA) kits for L-carnitine (KA0860, Abnova), retinol-binding protein (RBP, E90929Ra, Uscn), *β*2-microglobulin (*β*2-MG, E0260r, EIAab) and uromodulin (UMOD, E96918Ra, Uscn), and very low-density lipoprotein (VLDL, E1847r, EIAab) were used for the study. TRIzol reagent was obtained from Invitrogen (Carlsbad, CA, USA). M-MLV reverse transcriptase was obtained from Promega (Madison, WI, USA). The primers for all the genes were designed and synthesized by Generay Biotech (Shanghai, China). Polyvinylidene difluoride membrane was obtained from Millipore (Bed-ford, MA, USA). Primary antibodies including rabbit polyclonal antibodies against RST and OAT1 were provided by SaiChi Biotech (Beijing, P. R. China), MRP4 by Santa Cruz (CA, USA), ABCG2 by Cell Signaling Technology (Boston, MA, USA), OCTN2 by Abcam (Cambridge, MA, USA), CPT1 by Bioss Biotech (Beijing, P. R. China), and GAPDH by Jingmei Biotech (Shanghai, P. R. China).

### 2.2. Animals

Male Sprague-Dawley rats (7-week old, weighing 220–240 g) were purchased from the Laboratory Animal Center (Hangzhou, Zhejiang Province, P. R. China) and housed in plastic cages with a 12:12 h light-dark cycle at a constant temperature of 22–24°C. They were given standard chow *libitum *for study duration and allowed 1 week to adapt to laboratory environment before experiments. All procedures were carried out in accordance with Chinese legislation on the use and care of laboratory animals and with the guidelines established by the Institute for Experimental Animals of Nanjing University.

### 2.3. Experimental Protocol

Rats were randomly divided into 7 groups (*n* = 8 animals/group) as described below:

Group I: normal control. Rats were treated with saline (vehicle) by intragastric gavage (i.g.) at 8:00 AM and received saline (i.g.) at 2:00 PM;
Group II: rats were daily exposed to 3 mg/kg Cd at 8:00 am and received saline at 2:00 pm;
Group III: rats were daily exposed to 6 mg/kg Cd at 8:00 am and received saline at 2:00 pm;
Group IV: rats were daily exposed to 3 mg/kg Cd at 8:00 am and received 50 mg/kg quercetin at 2:00 pm;
Group V: rats were daily exposed to 3 mg/kg Cd at 8:00 am and received 100 mg/kg quercetin at 2:00 pm;
Group VI: rats were daily exposed to 6 mg/kg Cd at 8:00 am and received 50 mg/kg quercetin at 2:00 pm;
Group VII: rats were daily exposed to 6 mg/kg Cd at 8:00 am and received 100 mg/kg quercetin at 2:00 pm.

The doses of Cd were selected because that evidently induced changes in renal structure and function in rats [23, 24]. The doses of quercetin were selected because that showed protective effects on Cd-induced nephrotoxicity [22]. Furthermore, our preliminary experiments demonstrated hyperuricemia with dyslipidemia in 3 and 6 mg/kg Cd-exposed rats after 4 weeks, which were restored by the treatment of quercetin.

### 2.4. Urine, Blood, and Tissue Collection

At periodic intervals (the end of weeks 0, 1, 2, 3, and 4, resp.), rats were placed in metabolic cages individually for 24 h to collect urine over ice. Each urine sample was centrifuged at 3,000 × *g* (5 min, 4°C), and the volume was recorded. The supernatant was used for assays of NAG activity as well as UA, RBP, *β*2-MG, UMOD, ALB and protein levels. At the end of week 4, blood samples from rat's retroorbital venous plexus at 9:00-10:00 a.m. were centrifuged at 3,000 × *g* (5 min, 4°C) to get serum and then stored at 4°C for analyses of UA, Cd, Cr, L-carnitine, TG and VLDL levels, respectively. Then, rats were killed by decapitation, their kidney tissues were dissected quickly on ice and stored at −80°C for assays, respectively.

### 2.5. Determination of Biochemistry Parameters in Urine, Serum, and Kidney

Urine NAG activity, protein and ALB levels were measured using standard diagnostic kits, respectively. Serum, urine and renal L-carnitine, RBP, *β*2-MG and UMOD levels were measured using ELISA kits, respectively. UA levels in serum (Sur) and urine (Uur) were determined by the phosphotungstic acid method [25]. Cr levels in serum (Scr) and urine (Ucr) were determined spectrophotometrically using standard diagnostic kit (picric acid assay). Fractional excretion of UA (FE_UA_) is suggested to be a reliable indicator for renal UA excretion. This study calculated FE_UA_ using the formula: FE_UA_ = (Uur × Scr)/(Sur × Ucr) × 100, expressed as percentage. For TG assay, serum and kidney samples were determined using Van Handel-Caslson method. VLDL levels were measured using ELISA kit. Renal Na^+^-K^+^-ATPase activity was measured using standard diagnostic kit. For XO and XDH activity assays, renal cortex tissues were homogenized in 10 w/v 50 mM ice-cold potassium phosphate buffer (pH7.4) containing 5 mM ethylenediamine tetraacetic acid disodium salt and 1 mM phenylmethanesulfonyl fluoride (AMRESCO Inc, OH, USA) and centrifuged at 12,000 × *g* (15 min, 4°C). The supernatant fraction was centrifuged at 12,000 ×* g* (15 min, 4°C) once again and then used to detect XO and XDH activity by the method described previously [26].

### 2.6. RNA Isolation and Reverse Transcription-PCR

Total RNA was extracted from rat kidney using TRIzol reagent. The homogenate was mixed with 200 *μ*L chloroform and then centrifuged at 12,000 ×* g* for 15 min. Aqueous phase (about 0.5 mL upper layer) was precipitated with equal volume of isopropanol and centrifuging at 12,000 × *g* for 10 min. The final RNA total pellet was resuspended in 20 *μ*L DEPC water. Reverse transcription was performed with 1 *μ*g RNA using M-MLV reverse transcriptase for cDNA synthesis. PCR amplification was carried out using gene-specific PCR primers. The sequences of PCR primers were listed in Table 1. PCR products were electrophoresed on 1.2% agarose gels, visualized with Bio-Rad ChemiDoc XRS Gel Documentation system, and then quantified using Bio-Rad Quantity One 1D analysis software. Relative quantitation for PCR products was calculated by normalization to the amount of GAPDH mRNA levels.

### 2.7. Protein Preparation and Western Blot Analysis

Rat renal cortex was homogenized in 10 w/v buffer (10 mM Tris-HCl, 1 mM ethylenediaminetetra-acetic acid and 250 mM sucrose, pH 7.4, containing 15 *μ*g/mL aprotinin, 5 *μ*g/mL leupeptin, and 0.1 mM phenylmethyl sulfonyl fluoride), using a Polytron at setting 5 for 20 s, and centrifuged at 3,000 ×* g* for 15 min. The supernatant was centrifuged at 12,000 × *g *for 20 min. The final peptide samples were dissolved in Tris-HCl buffer (pH 7.5) containing 150 mM NaCl, 0.1% SDS, 1% NP-40, and 1% PMSF. After resolution of 75 *μ*g protein by 12% SDS-PAGE using Power Pac Basic electrophoresis apparatus (Bio-Rad, Hercules, CA, USA), protein samples were electrophoretically transferred onto PVDF membranes (Millipore, Shanghai, China), respectively. The membranes were blocked with 5% skim milk for 1 h and subsequently incubated with primary and secondary antibodies. Primary antibodies included rabbit polyclonal antibodies against RST (1 : 2000, NP_001030115), OAT1 (1 : 2000, NP_058920), MRP4 (1 : 1000, AAS78928.1) ABCG2 (1 : 1000, NP_852046.1), OCTN2 (1 : 200, NP_062142.1), CPT1(1 : 1000, NP_113747.2), and GAPDH (1 : 5000, NP_058704.1). Reactivity was detected using an anti-rabbit horseradish peroxidase-linked secondary antibody (1 : 1000). Immunoreactive bands were visualized via the Phototope-horseradish peroxidase Western Blot Detection System (Cell Signaling Technologies) and quantified via densitometry using Molecular Analyst software (Bio-Rad Laboratories, Hercules, CA, USA).

### 2.8. Histological Analyses

Rat kidney cortex was immediately fixed for 1 day at room temperature in 10% neutral buffered formalin for histopathological examination. Renal biopsies were dehydrated with a graded series of alcohol and embedded in paraffin. Specimens were cut in 7 *μ*m thick sections on a rotary microtome and mounted on APES-coated glass slides. Each section was deparaffinized in xylene, rehydrated in decreasing concentrations of alcohol in water, and stained with hematoxylin-eosin reagent (Sigma). The slide was mounted with neutral balsam. Another kidney cortex was snap-frozen immediately at −70°C. 6 *μ*m-thick cryostat sections were prepared on APES-coated glass slides. Each section was washed by distilled water and then stained with oil red O reagent (Sigma) for 5–10 min. After being washed with 60% isopropyl alcohol, the section was restained by hematoxylin.

### 2.9. Statistical Analysis

All data were expressed as mean ± SEM. Statistical analysis for experimental groups was performed by using a one-way analysis of variance followed by LSD post hoc test. *P* value < 0.05 was considered to be statistically significant. Figurers were obtained by GraphPad Prism 4 (GraphPad Software, Inc., San Diego, CA, USA).

## 3. Results

### 3.1. Body Weight and General Biomarkers of Nephropathy

In order to monitor the efficacy of Cd and subsequent quercetin treatment, body weight as well as urinary macromolecular enzyme activity and protein level was measured, respectively. As shown in Table 2, Cd exposure caused body weight reduction in rats (*P* < 0.001) compared with control group during the experimental period; however, quercetin treatment failed to restore this change.

**Figure 1:**
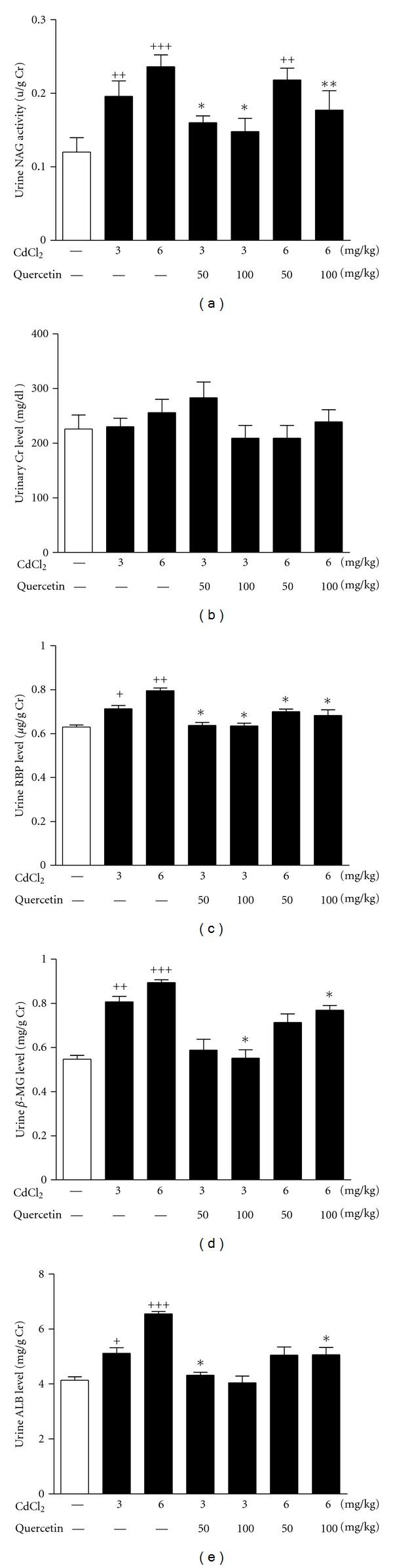
Effects of a 4-week treatment of CdCl_2_ and coadministration of quercetin on urinary activity of NAG (a), levels of Cr (b), RBP (c), *β*2-MG (d), and ALB (e) in rats. Values are mean ± SEM of *n* = 8 in each group. *P* value CdCl_2_ versus control at ^+^<0.05, ^++^<0.01, and ^+++^<0.001; treatment versus CdCl_2_ at *<0.05 and **<0.01 for LSD post hoc test.

Dysfunction and damage of renal tubules are characterized by the increased activity of urine NAG/Cr [23].  Figure 1(a) showed that Cd at 3 mg/kg (*P* < 0.01) and 6 mg/kg (*P* < 0.001) increased urine NAG activity in rats. Quercetin at 50 and 100 mg/kg significantly inhibited NAG activity (*P* < 0.05) in 3 mg/kg Cd-exposed rats, the latter decreased NAG activity (*P* < 0.01) in 6 mg/kg Cd-exposed rats. In addition, there were no significant changes of Cr levels in serum (data not shown) and urine (Figure 1(b)) among the tested groups.

As sensitive markers of macromolecular protein for renal tubular injury, urine levels of RBP (3 mg/kg: *P* < 0.05; 6 mg/kg: *P* < 0.01), *β*2-MG (3 mg/kg: *P* < 0.01; 6 mg/kg: *P* < 0.001), and ALB (3 mg/kg: *P* < 0.05; 6 mg/kg: *P* < 0.001) were significantly increased in rats after Cd exposure (Figures 1(c)-1(e)). Urine RBP and *β*2-MG levels in 3 and 6 mg/kg Cd-exposed rats were significantly decreased by the treatment of 100 mg/kg quercetin (*P* < 0.05), so were urine ALB in 6 mg/kg Cd-exposed rats. 50 mg/kg quercetin significantly decreased urine RBP levels (*P* < 0.05) in 3 and 6 mg/kg Cd-exposed rats and urine ALB levels (*P* < 0.05) in 3 mg/kg Cd-exposed rats (Figures 1(c)–1(e)).

**Figure 2:**
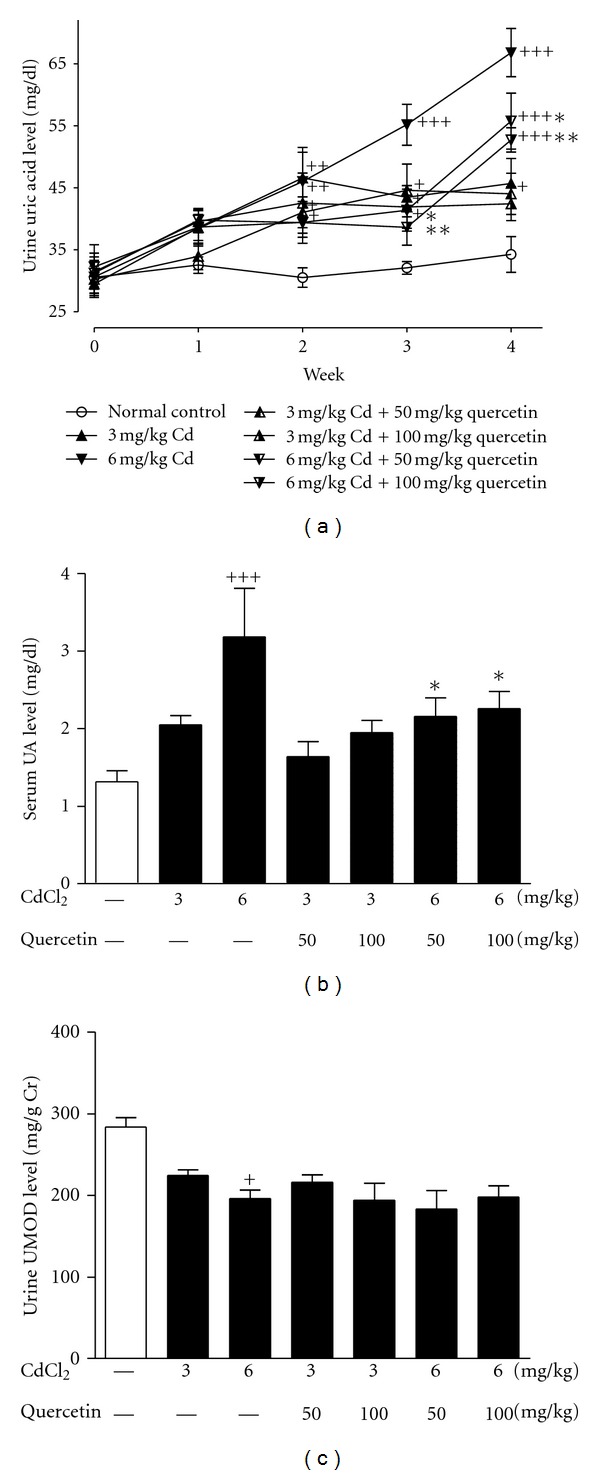
Effects of a 4-week treatment of CdCl_2_ and coadministration of quercetin on weekly urine UA levels (a), levels of serum UA (b), and urine UMOD (c) at the end of week 4. Values are mean ± SEM of *n* = 8 in each group. *P* value CdCl_2_ versus control at ^+^<0.05, ^++^<0.01, ^+++^<0.001; treatment versus CdCl_2_ at *<0.05 and **<0.01 for LSD post hoc test.

UA is a biomarker of nephropathy, and its detection is stable and easy. Compared with control group, urine UA levels were increased in rats exposed to Cd from week 2 and maintained until week 4 (3 mg/kg Cd: week 2, *P* < 0.01, week 3, *P* < 0.05, and week 4, *P* < 0.05; 6 mg/kg Cd: week 2, *P* < 0.01, week 3, *P* < 0.001, and week 4, *P* < 0.001) (Figure 2(a)). The decreased urine UA levels were observed in 6 mg/kg Cd-exposed rats receiving quercetin at week 4 (quercetin 50 mg/kg: *P* < 0.05; 100 mg/kg: *P* < 0.01). Furthermore, 6 mg/kg Cd exposure increased serum UA levels in rats compared with normal control (*P* < 0.001), which were significantly restored by the treatment of quercetin (*P* < 0.05) (Figure 2(b)). UMOD is a useful marker of renal dysfunction associated with hyperuricemia. The decreased urine UMOD levels (*P* < 0.05) were observed in 6 mg/kg Cd-exposed rats. Quercetin did not affect urine UMOD levels in Cd-exposed rats (Figure 2(c)).

**Figure 3:**
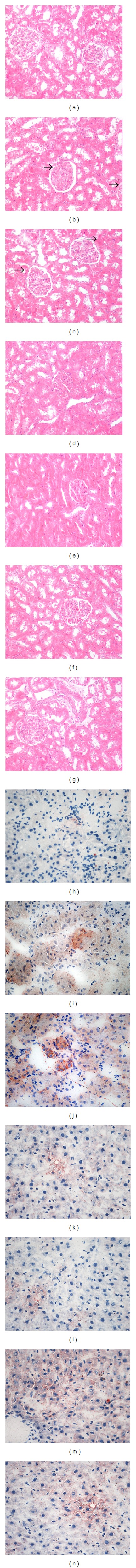
Renal cortex morphology in rats of a 4-week treatment of CdCl_2_ exposure and coadministration of quercetin. The kidney tissue slices were stained with hematoxylin or oil red O and then observed by microscope (original magnification ×200). Normal rat kidney (a) showed the identical structure of glomerulus and proximal tubules. CdCl_2_ exposure at 3 mg/kg (b) and 6 mg/kg (c) induced moderate inflammatory infiltration (black arrows). Quercetin at 50 mg/kg (d; f) and 100 mg/kg (e; g) significantly attenuated CdCl_2_-induced inflammatory infiltration around glomerulus and proximal tubules in kidney of rats. Normal rat kidney (h) showed no lipid deposition. CdCl_2 _exposure at 3 mg/kg (i) and 6 mg/kg (j) induced moderate lipid deposition (red). Quercetin at 50 mg/kg (k; m) and 100 mg/kg (l; n) significantly attenuated CdCl_2_-induced renal lipid deposition in renal tubular epithelial cells by oil red O-stain analysis.

Microscopically, 3 and 6 mg/kg Cd-induced mild inflammatory infiltration was observed in renal cortex of rats, which was remarkably ameliorated by the treatment of quercetin (Figures 3(a)–3(f)).

### 3.2. XOR Activity and Expression

**Figure 4:**
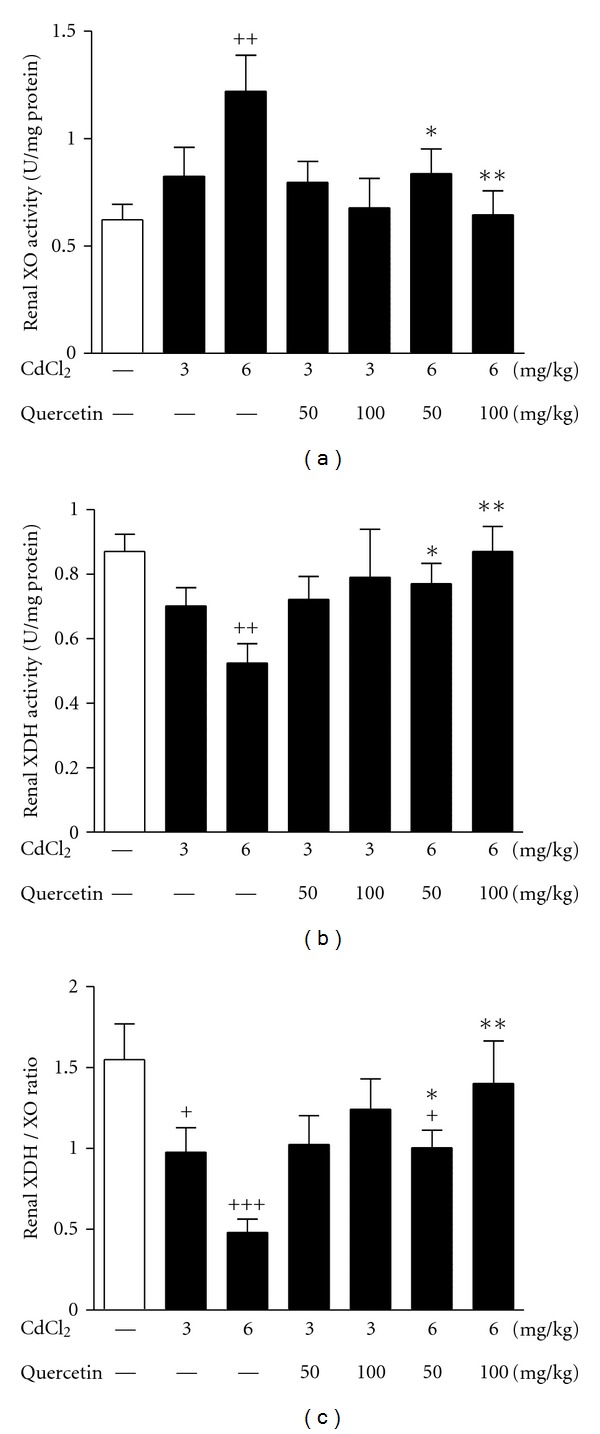
Effects of a 4-week treatment of CdCl_2_ and coadministration of quercetin on renal XO (a), XDH activity (b) and XDH/XO ratio in rats (c). Values are mean ± SEM of *n* = 6–8 in each group. *P* value CdCl_2_ versus control at ^+^<0.05, ^++^<0.01, and ^+++^<0.001; treatment versus CdCl_2_ at *<0.05 and **<0.01 for LSD post hoc test.

We next examined renal activity and expression of XOR, which plays an important role in UA synthesis. 6 mg/kg Cd exposure significantly increased renal activity of XO (*P* < 0.01) in rats compared with normal control, which were restored by quercetin at 50 mg/kg (*P* < 0.05) and 100 mg/kg (*P* < 0.01) (Figure 4(a)). However, 6 mg/kg Cd exposure relatively decreased renal activity of XDH (*P* < 0.01) (Figure 4(b)) with significantly decreased XDH/XO ratio (*P* < 0.001) (Figure 4(c)), which were restored by quercetin treatment. Both Cd exposure and quercetin treatment failed to significantly alter renal XDH mRNA levels in rats (data not shown).

### 3.3. FE_**UA**_

**Figure 5:**
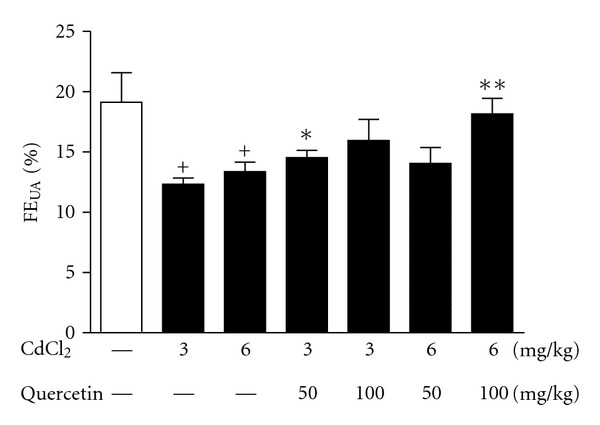
Effects of a 4-week treatment of CdCl_2_ and coadministration of quercetin on kidney handling fractional excretion of UA (FE_UA_) in rats. Values are mean ± SEM of *n* = 8 in each group. *P* value CdCl_2_ versus control at ^+^<0.05; treatment versus CdCl_2_ at *<0.05 and **<0.01 for LSD post hoc test.

FE_UA_ is investigated with the unbalanced bidirectional transport of UA in renal proximal tubules, supporting the predominant mechanism for hyperuricemia and renal UA underexcretion [19, 27]. FE_UA_ in 3 and 6 mg/kg Cd-exposed rats were significant lower than that of normal control (*P* < 0.05) (Figure 5). Quercetin significantly increased FEUA in rats exposed to Cd at 3 mg/kg (50 mg/kg: *P* < 0.05) and 6 mg/kg (50 mg/kg: *P* < 0.01; 100 mg/kg: *P* < 0.001), suggesting that quercetin may enhance renal UA excretion.

### 3.4. Expression of UA Transport-Related Proteins and Activity of Na^+^-K^+^-ATPase

**Figure 6:**
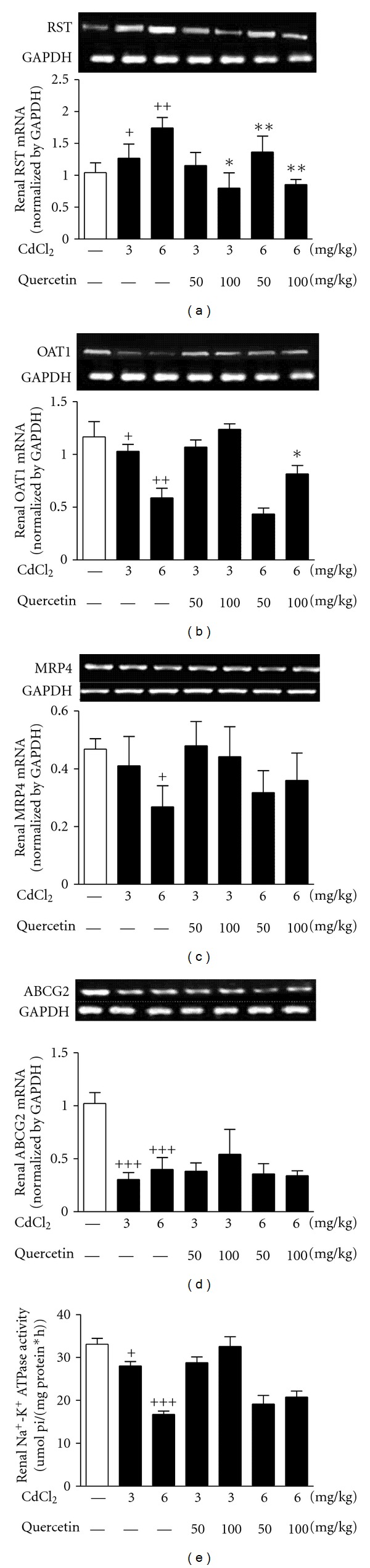
Effects of a 4-week treatment of CdCl_2_ and coadministration of quercetin on expression of RST (a), OAT1 (b), MRP4 (c), and ABCG2 (d) at mRNA levels and activity of Na^+^-K^+^-ATPase (e) in renal cortex of rats. The mRNA levels were normalized by GAPDH. Values are mean ± SEM of *n* = 4–6 in each group. *P* value CdCl_2_ versus control at ^+^ < 0.05, ^++^ < 0.01, and ^+++^ < 0.001; treatment versus CdCl_2_ at *<0.05 and **<0.01 for LSD post hoc test.

**Figure 7:**
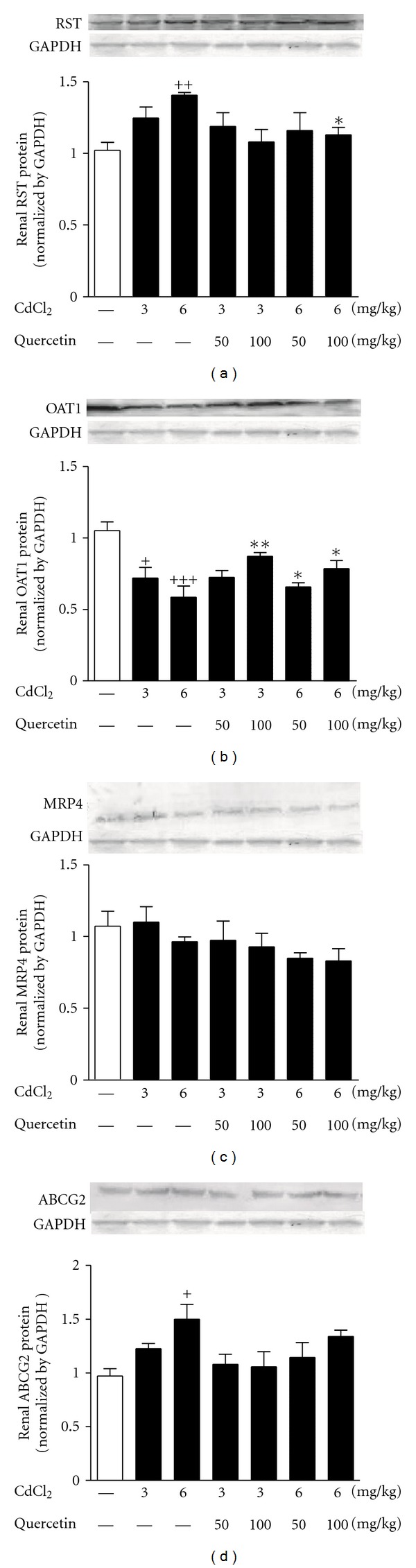
Effects of a 4-week treatment of CdCl_2_ and coadministration of quercetin on expression of RST (a), OAT1 (b), MRP4 (c), and ABCG2 (d) at protein levels in renal cortex of rats. The protein levels were normalized by GAPDH. Values are mean ± SEM of *n* = 4–6 in each group. *P* value CdCl_2_ versus control at ^+^<0.05, ^++^<0.01, ^+++^<0.001; treatment versus CdCl_2_ at *<0.05, and **<0.01 for LSD post hoc test.

In order to explore the reasons for renal UA excretion abnormality, we examined the expression levels of renal RST, OAT1, MRP4, and ABCG2 in Cd-exposed rats by RT-PCR and Western blot analyses, respectively. As shown in Figures 6 and 7, Cd exposure significantly increased RST mRNA (3 mg/kg: *P* < 0.05; 6 mg/kg: *P* < 0.01) (Figure 6(a)) and protein (3 mg/kg: *P* < 0.05; 6 mg/kg: *P* < 0.001) (Figure 7(a)) levels and decreased OAT1 mRNA (3 mg/kg: *P* < 0.05; 6 mg/kg: *P* < 0.01) (Figure 6(b)) and protein (6 mg/kg: *P* < 0.01) levels (Figure 7(b)) in the kidney of rats compared with normal control. Cd exposure suppressed renal mRNA levels of MRP4 (6 mg/kg: *P* < 0.05) (Figure 6(c)) and ABCG2 (3 and 6 mg/kg: *P* < 0.001) (Figure 6(d)) and increased renal protein levels of ABCG2 (6 mg/kg: *P* < 0.05) (Figure 7(d)) in rats. Moreover, 100 mg/kg quercetin ameliorated 3 mg/kg Cd-induced changes of RST mRNA levels (*P* < 0.05) in rats (Figure 6(a)). Meanwhile, quercetin ameliorated 6 mg/kg Cd-induced changes of RST mRNA levels (50 and 100 mg/kg: *P* < 0.01) (Figure 6(a)) and protein levels (100 mg/kg: *P* < 0.05) (Figure 7(a)) in rats. The changed expression levels of renal OAT1 mRNA (100 mg/kg: *P* < 0.05) and protein (50 and 100 mg/kg: *P* < 0.05) in 6 mg/kg Cd-exposed rats were also restored by the treatment of quercetin (Figures 6(b) and 7(b)). However, quercetin performed no effects on renal MRP4 and ABCG2 in Cd-exposed rats.

Na^+^-K^+^-ATPase is an energy supplier for some of UA transport-related proteins such as OAT1 [28]. Cd exposure significantly decreased renal Na^+^-K^+^-ATPase activity in rats compared with normal control (3 mg/kg: *P* < 0.05; 6 mg/kg: *P* < 0.001) (Figure 6(e)). However, quercetin failed to affect the activity in Cd-exposed rats.

### 3.5. Serum and Renal TG and VLDL Levels

**Figure 8:**
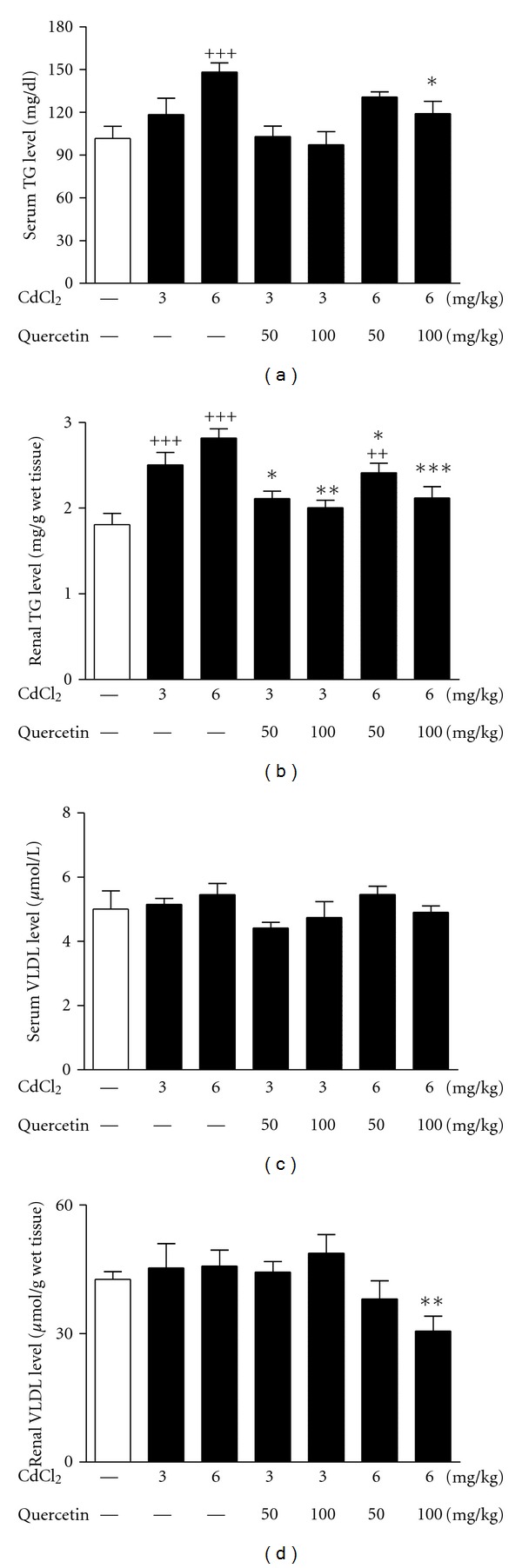
Effects of a 4-week treatment of CdCl_2_ and coadministration of quercetin on TG levels in serum (a) and kidney (b); VLDL levels in serum (c) and kidney (d) in rats. Values are mean ± SEM of *n* = 8 in each group. *P* value CdCl_2_ versus control at ^ ++^<0.01 and ^+++^<0.001; treatment versus CdCl_2_ at *<0.05, **<0.01, and ***<0.001 for LSD post hoc test.

UA and XOR are confirmed to be related to lipid metabolism [13], we addressed the question whether Cd nephrotoxicity was correlated to renal lipid metabolism disorder. Thus, TG and VLDL levels were detected in Cd-exposed rats. Cd exposure increased TG levels in serum (6 mg/kg: *P* < 0.001) and kidney (3 and 6 mg/kg: *P* < 0.001) of rats compared with normal control (Figures 8(a) and 8(b)), exhibiting renal lipid accumulation. 100 mg/kg quercetin significantly reduced serum TG levels (*P* < 0.05) in 6 mg/kg Cd-exposed rats (Figure 8(a)). The increased renal TG levels were ameliorated by the treatment of quercetin at 50 mg/kg (*P* < 0.05) and 100 mg/kg (*P* < 0.01) in 3 and 6 mg/kg Cd-exposed rats (Figure 8(b)). However, Cd exposure did not significantly change serum and renal VLDL levels (Figures 8(c) and 8(d)). 100 mg/kg quercetin reduced renal VLDL levels (*P* < 0.01) in 6 mg/kg Cd-exposed rats (Figure 8(d)).

Moreover, oil red staining analysis revealed moderate lipid deposition observed in tubular epithelial cells in renal tissue sections of Cd-exposed rats, which could be improved by the treatment of quercetin (Figures 3(h)–3(n)).

### 3.6. L-Carnitine Levels and Expression of Renal Lipid Metabolism-Related Genes

**Figure 9:**
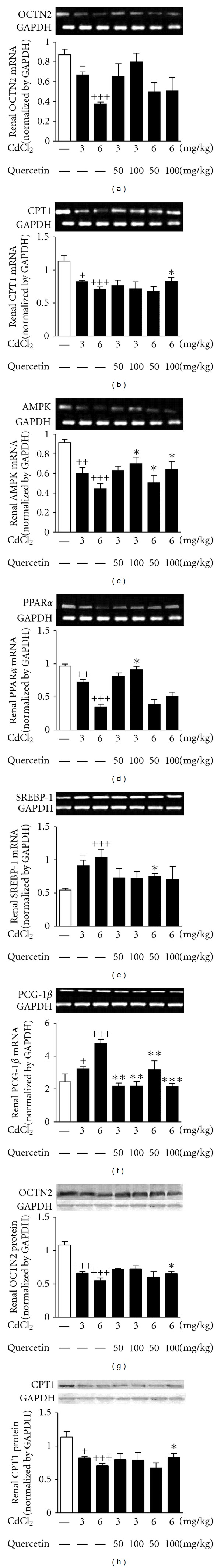
Effects of a 4-week treatment of CdCl_2_ and coadministration of quercetin on expression of OCTN2 (a), CPT1 (b), AMPK (c), PPAR*α* (d), SREBP-1 (e), PGC-1*β* (f) at mRNA levels, and OCTN2 (g) and CPT1 (h) at protein levels in renal cortex of rats. The mRNA levels or protein levels were normalized by GAPDH, respectively. Values are mean ± SEM of *n* = 4–6 in each group. *P* value CdCl_2_ versus control at ^+^ < 0.05, ^++^ < 0.01, and ^+++^<0.001; treatment versus CdCl_2_ at *<0.05, **<0.01, and ***<0.001 for LSD post hoc test.

L-carnitine is essential to fatty acid *β*-oxidation from mitochondrial membrane mediated by CPT1. OCTN2 is an important transporter for L-carnitine reabsorption [29]. However, no significant changes of L-carnitine levels in serum, urine, and kidney cortex were observed in the tested groups (data not shown). Cd exposure reduced renal levels of OCTN2 and CPT1 mRNA (3 mg/kg: *P* < 0.05; 6 mg/kg: *P* < 0.001) (Figures 9(a) and 9(b)) and protein (*P* < 0.001) (Figures 9(g) and 9(h)) in rats compared with normal control. These data indicated that Cd-induced OCTN2 downregulation did not affect L-carnitine levels, which might not be an important factor to cause renal lipid metabolism disorder in Cd nephrotoxicity of rats.

Next, we analyzed whether Cd exposure affected the expression levels of other lipid metabolism-related genes in the kidney of rats. Compared with normal control, Cd exposure significantly suppressed renal mRNA levels of AMPK (Figure 9(c)) and PPAR*α* (Figure 9(d)) (3 mg/kg: *P* < 0.01; 6 mg/kg: *P* < 0.001). Cd-induced elevation in renal mRNA levels of and SREBP-1 (Figure 9(e)) and PGC-1*β* (Figure 9(f)) (3 mg/kg: *P* < 0.05; 6 mg/kg: *P* < 0.001) was observed in rats.

Quercetin treatment increased AMPK in rats exposed to Cd at 3 mg/kg (100 mg/kg: *P* < 0.05) and 6 mg/kg (50 and 100 mg/kg: *P* < 0.05) (Figure 9(c)). Quercetin at 100 mg/kg also ameliorated 3 mg/kg Cd-induced downregulation of renal PPAR*α* mRNA levels (*P* < 0.05) (Figure 9(d)) as well as 6 mg/kg Cd-induced downregulation of renal OCTN2 protein levels (Figure 9(g)), CPT1 mRNA, and protein levels (*P* < 0.05) in rats (Figures 9(b) and 9(h)). Moreover, quercetin significantly downregulated renal SREBP-1 (50 mg/kg: *P* < 0.05) and PGC-1*β* (100 mg/kg: *P* < 0.01) (Figures 9(e) and 9(f)) in Cd-exposed rats.

## 4. Discussion

The main findings of this study were that Cd exposure induced renal UA transport system dysfunction with XOR hyperactivity and impaired renal AMPK-PPAR*α*/PGC-1*β* signal pathway, resulting in renal lipid accumulation involved in Cd nephrotoxicity of rats. By administering a dietary flavonoid quercetin to Cd-exposed rats, it ameliorated renal UA transport system dysfunction with XOR hyperactivity and subsequently improved renal AMPK-PPAR*α*/PGC-1*β* signal pathway impairment to restore disorder of renal lipid metabolism, exhibiting its nephroprotection.

The kidney is the primary critical target of toxicity, where Cd accumulation reaches the threshold [23, 30–32], leading to pathological damaged levels of urine enzyme (NAG) and proteins (RBP, *β*2-MG, ALB, and UMOD) observed in the present study. Quercetin treatment restored Cd-induced renal dysfunction and toxicity in rats.

In parallel with urine enzyme hyperactivity, a continued rise of serum UA levels was observed in Cd-exposed rats. The UA change could take place in the early stage of Cd exposure, as the urine UA levels increased significantly from week 2. The kidney is a target organ for Cd; therefore, it was necessary to investigate the effects of Cd on renal XOR activity and UA transport-related proteins in rats. Being consistent with activated renal XO in Cd-exposed Swiss albino mice [33, 34], the present study confirmed activation of renal XO in Cd-exposed Sprague-Dawley rats with a significant reduction of renal XDH/XO ratio, which resulted from the increased XO activity with the relatively decreased XDH activity. These data further demonstrates that Cd enhances the conversion of XDH to XO [7] in the kidney of rats, possibly causing serious renal damage induced by XOR hyperactivity-mediated ROS. Quercetin, as a XOR inhibitor [20, 35, 36], inhibits XOR activation in the kidney of ischemia-reperfusion rat [36]. In the present study, quercetin was confirmed to prevent the conversion of renal XDH to XO, which were consistent with its attenuation of Cd-induced hyperuricemia and renal injury.

More importantly, 6 mg/kg Cd exposure was found to upregulate RST and downregulate OAT1, MRP4, and ABCG2 in the kidney of rats, indicating that Cd exposure may alter renal function for UA transport. Na^+^-K^+^-ATPase is the energy supplier for some organic ion transporters such as OAT1 [28]. The altered Na^+^-K^+^-ATPase activity is incriminated to play a role in renal tubular syndrome associated with Cd-induced nephrotoxicity [37, 38]. Hypoactivity of renal rNa^+^-K^+^-ATPase was observed in the present study. Therefore, renal UA transport system dysfunction with XOR hyperactivity may be involved in the mechanisms of Cd-induced hyperuricemia and renal dysfunction in rats. Our previous study showed that quercetin restored fructose-induced dysexpression of renal RST and OAT1 in hyperuricemic rats [19]. In this study, although no effect on renal Na^+^-K^+^-ATPase activity, quercetin reduced serum UA levels, possibly through its amelioration of Cd-induced abnormality of renal RST and OAT1 to enhance renal UA excretion, resulting in relief of hyperuricemia and kidney dysfunction in rats. Thus, renal UA transport system is suggested to be target for quercetin's action in Cd nephrotoxicity.

As important cross-regulators, UA and XOR are associated with lipid metabolism [13]. XOR inhibitors allopurinol and quercetin are confirmed to prevent fructose-induced hypertriglyceridemia in rats [19, 39]. Lipids play an important role in the progression and development of kidney diseases [14]. The present study demonstrated that Cd induced moderate lipid accumulation and deposition in renal cortex of rats with high TG levels, which were restored by the treatment of quercetin. These results indicate that alteration of renal UA transport system with XOR hyperactivity may be associated with renal lipid metabolism disorder of Cd nephrotoxicity in rats.

Lipid metabolism regulator PPAR*α* and its target genes OCTN2 and CPT1 are involved in mitochondrion fatty acid *β*-oxidation, playing an important role in nonadipose tissue [40]. PPAR*α* can protect renal tubular cells from doxorubicin-induced ROS [41]. Its agonists prevent renal oxidative stress and damage to improve proteinuria in hypertensive patients with renal disease [42]. Furthermore, XOR activation and ROS increase expression of PGC-1 [43], which is suggested to enhance PPAR*α*-mediated transcriptional activity [44, 45]. Hepatic PGC-1*β* overexpression reduces the beneficial effects of PPAR*α* activation on gene expression, leading to hyperlipidemia [45]. In addition, PGC-1*β* activates expression of lipogenic genes via direct coactivation of SREBP-1, a major regulator of fatty acids synthesis [46]. Interestingly, the present study found that Cd exposure decreased PPAR*α*, OCTN2, and CPT1 and increased PGC-1*β* expression, which possibly activated SREBP-1 in the kidney of rats. These results indicate that renal downregulation of PPAR*α* and its target genes mediated by PGC-1*β* overexpression may be involved in renal reduction of fatty acid *β*-oxidation and disorder of lipid metabolism in Cd-exposed rats with renal UA transport function impairment with XOR hyperactivity.

It is well known that AMPK regulates downstream PPAR*α* to affect the transcription of numerous genes including OCTN2 and CPT1 [47]. AMPK activation enhances fatty acid *β*-oxidation in skeletal muscle by activating PPAR*α* and PGC-1 [48]. Additionally, SREBP-1 is negatively regulated by AMPK. Interestingly, renal AMPK expression was downregulated in Cd-exposed rats in the present study. PPAR*α* and its target genes are involved in cross-talking of lipid metabolism with oxidative stress by Cd-induced UA overproduction and ROS synthesis. Thus, the ability of Cd to affect renal lipid metabolism-related AMPK-PPAR*α*/PGC-1*β* signal pathway possibly mediated by renal UA transport function impairment with renal XO hyperactivity may have significant implication for the pathophysiology of Cd-induced renal injury in rats. The precise mechanisms need to be further explored in suitable cell models.

Quercetin, as an activator of AMPK [49], was confirmed to upregulate AMPK, PPAR*α*, CPT1, and OCTN2, as well as downregulate PGC-1*β* and SREBP-1 in the kidney of Cd-exposed rats, which were parallel with its restoration of renal lipid accumulation. Thus, quercetin with regulation of renal UA transport system and XOR activity may reduce renal lipid accumulation partly mediated by improving renal AMPK-PPAR*α*/PGC-1*β* signal pathway impairment in Cd nephrotoxicity of rats.

## 5. Conclusion

This study demonstrated that Cd-induced UA excretion dysfunction with excess synthesis further aggravated UA congestion and made renal lesion more serious in rats. Abnormality of renal UA transport system with XOR activity may be a key target for disorder of renal lipid metabolism and induction of secondary renal damage process in rats exposed to Cd. This study was the first to focus, and confirm the relative importance of renal UA transport system dysfunction with XOR activation and AMPK-PPAR*α*/PGC-1*β* signal pathway impairment involved in Cd nephrotoxicity of rats. Quercetin was found to ameliorate renal UA transport system dysfunction with XOR hyperactivity and improve renal AMPK-PPAR*α*/PGC-1*β* signal pathway impairment and subsequently reduce renal lipid accumulation in rats. Quercetin may serve as antihyperuricemic and antidyslipidemic agent to prevent Cd-evoked nephrotoxicity.

## Figures and Tables

**Table 1:** Summary of the sequences of RT-PCR primers, the appropriate annealing temperature used in experiments, and product size.

Genes	Primer	Annealing temperature (°C)	Product size (bp)
GAPDH	S 5′-TCAACGGCACAGTCAAGG-3′	54	299
	A 5′-ACCAGTGGATGCAGGGAT-3′		
RST	S 5′-CACAGTGGGCAGACTGGACCAGAGC-3′	57	412
	A 5′-CCAAGGATGAGCGAAGGA-3′		
OAT1	S 5′-TAATACCGAAGAGCCATACGA-3′	56	358
	A 5′-TCCTGCTGCTGTTGATTCTGC-3′		
MRP4	S 5′-AAATCGGAATCTCCTGTCTG −3′	56	203
	A 5′-TATGAGGTCGGCGAATGA-3′		
ABCG2	S 5′- TAGCAGCAAGGAAAGAC−3′	54	835
	A 5′-TGATGACAGAACGAGGTA-3′		
XDH	S 5′-CTTTGCGAAGGATGAGGTT-3′	58	412
	A 5′-CACTCGGACTACGATTCTGTT-3′		
CPT1	S 5′-CCACGAAGCCCTCAAACAGA-3′	57	315
	A 5′-AGCACCTTCAGCGAGTAGCG-3′		
OCTN2	S 5′-AGGTTTGGTCGCAAGAATG-3′	56	458
	A 5′-AACTCACTGGGATCGAAGAT-3′		
PPAR*α*	S 5′-GGCTCGGAGGGCTCTGTCATC-3′	56	655
	A 5′-ACATGCACTGGCAGCAGTGGA-3′		
SREBP-1	S 5′-GGAGCGAGCATTGAACTGTAT-3′	58	344
	A 5′-GGGCAGCCTTGAAGGAGTA-3′		
PGC-1*β*	S 5′-GGTACAGCTCATTCGCTACAT-3′	58	210
	A 5′-TAGGGCTTGCTAACATCACA-3′		

**Table 2:** Effects of a 4-week treatment of cadmium chloride (CdCl_2_) and coadministration of quercetin on body weight (g) in rats. (Values are mean ± SEM from eight rats in each group).

Group	Body weight (g)
Normal control	352.4 ± 11.9
3 mg/kg CdCl_2_	323.0 ± 18.5^+^
3 mg/kg CdCl_2_ + 50 mg/kg quercetin	309.8 ± 9.3^+++^
3 mg/kg CdCl_2_ + 100 mg/kg quercetin	309.7 ± 9.3^+++^
6 mg/kg CdCl_2_	305.4 ± 18.5^+++^
6 mg/kg CdCl_2_ + 50 mg/kg quercetin	309.3 ± 14.1^+++^
6 mg/kg CdCl_2_ + 100 mg/kg quercetin	310.1 ± 7.6^+++^

*P* value CdCl_2_ versus control at ^+^<0.05 and ^+++^<0.001.
